# Development and Prospect of Vacuum High-Pressure Gas Quenching Technology

**DOI:** 10.3390/ma16237413

**Published:** 2023-11-29

**Authors:** Shengde Hu, Lin Zhu, Mao Zhang, Xuefeng Tang, Xinyun Wang

**Affiliations:** 1 School of Materials and Metallurgy, Wuhan University of Science and Technology, Wuhan 430081, China; adhello@163.com (S.H.); zzmocuishle@163.com (L.Z.); 2 State Key Laboratory of Materials Processing and Die & Mould Technology, School of Materials Science and Engineering, Huazhong University of Science and Technology, Wuhan 430074, China; zhangmao@hust.edu.cn

**Keywords:** vacuum gas quenching, temperature field, high pressure flow field, vacuum heat treatment, high-precision simulation

## Abstract

As industrial modernization surges forward, the heat treatment industry strives for lower pollution, reduced oxidation and defects, minimized waste, and automatization. This paper reviews the mechanisms, processes, equipment, and simulations of the vacuum gas quenching technology, presenting a comprehensive account of the structure and working principle of a typical vacuum gas quenching furnace. Firstly, the mechanism of the heat transfer process, flow process, and flow–heat transfer–phase transition coupling were summarized. Then, the influences of process parameters on the mechanical properties and distortion of vacuum gas quenched workpieces, as well as the process optimization methods, were discussed. Finally, the advantages of vacuum gas quenching in energy saving, low pollution, and high efficiency were introduced, with the future development directions figured out.

## 1. Introduction

The liquid medium quenching cooling process consists of three distinct stages—steam film, boiling, and convection—each of which exhibits unique heat transfer coefficients. This leads to significant thermal stress and large temperature gradients during cooling, often resulting in distortion of the workpiece. The development of electric vacuum technology led to the emergence of vacuum heat treatment in the early time, and due to the limitation of the equipment’s technical level, this technology did not progress significantly for a long time. After that, advanced equipment such as air-cooled vacuum heat treatment furnaces, cold wall vacuum oil quenching furnaces, and vacuum heating high-pressure gas quenching furnaces were introduced, paving the way for further advancements in vacuum heat treatment. In recent years, vacuum heat treatment has become one of the most widely used advanced heat treatment techniques. The United States has planned to achieve the highest utilization rate of heat treatment energy and the lowest environmental pollution by 2020. Likewise, other developed nations have also put forth their own strategies to reduce production costs and processing time. In China, the pursuit of independent research, development, and manufacturing capabilities has become a national priority as the country undergoes industrial transformation and development. Leading manufacturers are investing in the development of new heat treatment technologies, presenting both opportunities and challenges for the industry.

Vacuum gas quenching is a heat treatment technology that involves heating a metal workpiece under negative pressure (below 0.1 MPa) and then quenching it with high-pressure and high-speed flowing gas. Compared to traditional oil quenching and salt bath quenching, vacuum high-pressure gas quenching technology offers several advantages [1,2]: (1) No oxidation or decarburization occurs during vacuum heating, resulting in clean surfaces without a metamorphic layer. The distortion of the workpiece is small, and its comprehensive performance is excellent. (2) It does not pollute the environment, eliminating the need for waste treatment. (3) It enables precise control of heating and cooling thanks to highly accurate furnace temperature measurement and monitoring. (4) It has a high degree of automation, significantly reducing the operator’s labor intensity. Therefore, vacuum high-pressure gas quenching technology, as one of the high-efficiency, energy-saving, and clean heat treatment technology, has been a hot topic in the development of heat treatment technology in recent decades. Advanced industrial countries such as the United States, Germany, and Japan have made it their leading heat treatment process for high-strength steel (HSS) and high alloy die steel. The 2 MPa ultra-high pressure gas quenching furnace with a mixture of helium and nitrogen gas has reached or approached the oil cooling level when processing large-section workpieces. The 4 MPa H_2_ quenching can achieve the water cooling level under laboratory conditions [3].

## 2. Development of Vacuum High-Pressure Gas Quenching Technology

### 2.1. Development of Vacuum High-Pressure Gas Quenching Equipment

Vacuum heat treatment was first invented in the 1920s and is mainly used in the annealing and aging of metals [4]. In cases where high surface quality is required, additional processes such as tempering and precipitation hardening may still be required after vacuum quenching and solution heat treatment. When quenching HSS using ordinary air electric furnaces and salt furnaces, a thick decarburizing oxide layer will be produced, requiring the workpiece to be sent back to the workshop for finishing. This process is not only complicated and time-consuming, but also causes severe equipment wear. Therefore, protective atmosphere furnaces and vacuum furnaces are often preferred for quenching. Zhou et al. [5,6] carried out atmosphere protection heat treatment and vacuum quenching heat treatment on 30CrMnSiNi2A steel. At the same quenching and tempering temperatures, the depth of the decarburization layer of the sample in the atmosphere protection furnace was 0.1 mm, while there was no oxidation or decarburization on the surface of the steel after vacuum quenching.

Vacuum gas quenching was first invented in the 1970s [7]. This process involves heating the workpiece within a vacuum environment followed by rapid cooling within a chamber filled with high-purity reactive gas (such as N_2_). The vacuum gas quenching furnace with low early gas quenching pressure is suitable for materials with low critical cooling rates of martensite, such as HSS and high carbon and high chromium steel. Unlike other cooling methods, gas quenching does not induce a physical state change, resulting in a more uniform heat transfer and reduced risk of distortion [8]. To allow high-alloy tool steel with a large size to obtain a superior thermal effect through vacuum treatment, vacuum gas quenching was developed, and a new gas quenching process with cooling gas pressure up to (0.5–0.7) MPa was proposed. The crux of popularizing vacuum heat treatment technology lies in the equipment. As shown in Table 1, in 1975, the world’s first vacuum high-pressure gas quenching furnace was developed by Ipsen Company in Germany [3]. This furnace adopted a high-power two-speed turbine fan, enabling a charging pressure of 0.5 MPa, and also included a heat exchanger and a flow guide for optimizing the flow rate and turbulence for optimal quenching uniformity. In addition, microprocessors and program cycle controllers, as well as temperature and vacuum control instruments were also integrated into the furnace. This furnace was capable of automatically selecting a cooling mode, and this new gas quenching vacuum furnace was used for heat treatment of aerospace parts, HSS tools, hot work die steel, high-alloy tool steel, and stainless steel parts [9]. In 1980, the Japanese Vacuum Technology Company used an NVFC series high-pressure vacuum heat treatment furnace produced by German Ipsen Company to tested the gas quenching of SKH-9 (M2) and SKD-11 (D2). Meanwhile, since the 1980s, vacuum heat treatment technology has developed rapidly in China. The introduction of advanced equipment has led to the wide adoption of vacuum heat treatment technology. In 1981, a ZC-30, a ZC-65 vacuum quenching furnace and a ZCT-65 vacuum carburizing furnace were introduced and installed in the Heat Treatment Institute of Chinese Tianjin Machinery Research and Chinese Design Institute. Compared with salt bath heat treatment, vacuum heat treatment has increased the service life of the workpiece by more than one time, even dozens of times, and achieved superior economic benefits [10]. In 1983, Shenyang Institute of Vacuum Technology of the Ministry of Mechanical and Electrical Engineering developed the first pressurized vacuum gas quenching furnace in China [11]. The furnace increases the gas pressure in the furnace from negative pressure to positive pressure of 0.1 MPa, thus improving the cooling capacity of the domestic vacuum quenching furnace. However, these furnaces are still not enough to meet the heat treatment requirements of workpieces with large cross-sections and complex shapes. 

In 1988, Poland Erte Company independently designed and manufactured a 0.5 MPa gas quenching vacuum furnace (VDN-513R). Compared to a HSS drill produced by a salt bath, the cutting life of a HSS drill produced by vacuum gas quenching using the Poland VDN-513R vacuum furnace was increased by two to three times, while the production efficiency improved threefold [12]. In the same year, Chinese Shenyang Institute of Vacuum Technology made the first high-pressure vacuum quenching furnace in China, which can be pressurized to 0.5 MPa. By the end of the 20th century, SECO/WARWICK developed vacuum gas quenching furnaces of 0.2, 0.6, 0.8, 1, and 2 MPa, with vertical and horizontal types [13]. German ALD Vacuum Industries Co., Ltd. developed a new generation of two-chamber vacuum furnaces called DualTherm [14]. The two-chamber vacuum furnace comprised a treatment chamber and an independent gas quenching chamber. The two chambers were connected by a transport chamber through which workpieces were loaded and removed from the furnace. The vacuum state of the heating chamber can be maintained in the double-chamber vacuum furnace, enabling energy savings and improved production efficiency by allowing only the workpiece to be cooled while the hot furnace walls remain uncooled. Subsequently, production lines from single-chamber vacuum furnaces and double-chamber vacuum furnaces to integrated continuous vacuum furnaces had been developed for heat treatment for a variety of purposes. At this time, a team of gas quenching furnace design and manufacturing has been formed in China. Chinese Shenyang Vacuum Institute, Chinese Beijing Huaxiang Company and Chinese Capital Aerospace Machinery Factory have been able to provide 5 × 10^5^ Pa gas quenching vacuum furnaces [15]. Shenyang Vacuum Technology Research Institute has developed a new generation of high-pressure vacuum gas quenching furnaces, which not only share the advantages of ordinary vacuum gas furnaces (good surface brightness of heat-treated products, small distortion, and no secondary cleaning), but also greatly expand the range of materials and section size of treated workpieces [16]. 

In 2001, German ALD Company introduced the ModulTherm, a modular system that connects up to 10 independent vacuum treatment chambers via a moving assembly that contains a transfer chamber and a quenching chamber on a track [14]. In 2013, in order to solve the shortage of low-alloy steel parts, large-size molds and cutters and high-density loaded workpieces in the actual production of vacuum high-pressure gas quenching, Chinese Beijing Hu Axiang Electric Furnace Technology Co., Ltd. developed a vertical gas quenching vacuum furnace with 1 MPa high pressure and large flow, and adopted a number of national patent technologies, with the loading capacity increased to 2500 kg [17].

### 2.2. Structure and Working Principle of Vacuum Gas Quenching Resistance Furnace

The vacuum gas quenching furnaces are typically structured as horizontal single-chamber structures, horizontal double-chamber structures, and vertical structures. The horizontal single-chamber structure is mainly composed of a furnace body, heating chamber, strong cooling system, vacuum system, water cooling system, and other parts [18]. Its working principle is that after vacuuming, the workpiece is heated to the quenching temperature and filled with high-pressure inert gas for rapid cooling. To speed up the cooling procedure, fans are used for a high flow rate of gas circulation to meet the requirements of the microstructure and performance after quenching. Generally, the gas quenching vacuum furnaces with the horizontal single-chamber structure use graphite heaters and hard graphite felt for heat insulation. Gas quenching vacuum furnace provides a visual operation interface and automatic switch, enabling automatic control. The horizontal single-chamber vacuum gas quenching furnace is usually controlled by PLC, which offers strong anti-interference capabilities and stable program operation, making it especially suitable for use in strong interference environments. The heating and temperature control system adopts the voltage regulator control mode, which has the advantages of simple control, reliable operation, and low cost. Additionally, safety measures such as short circuit protection are added to ensure the safe and stable operation of the system [19]. To ensure the safe and reliable operation of the system, self-locking and interlocking are adopted in both software and hardware.

**Figure 1:**
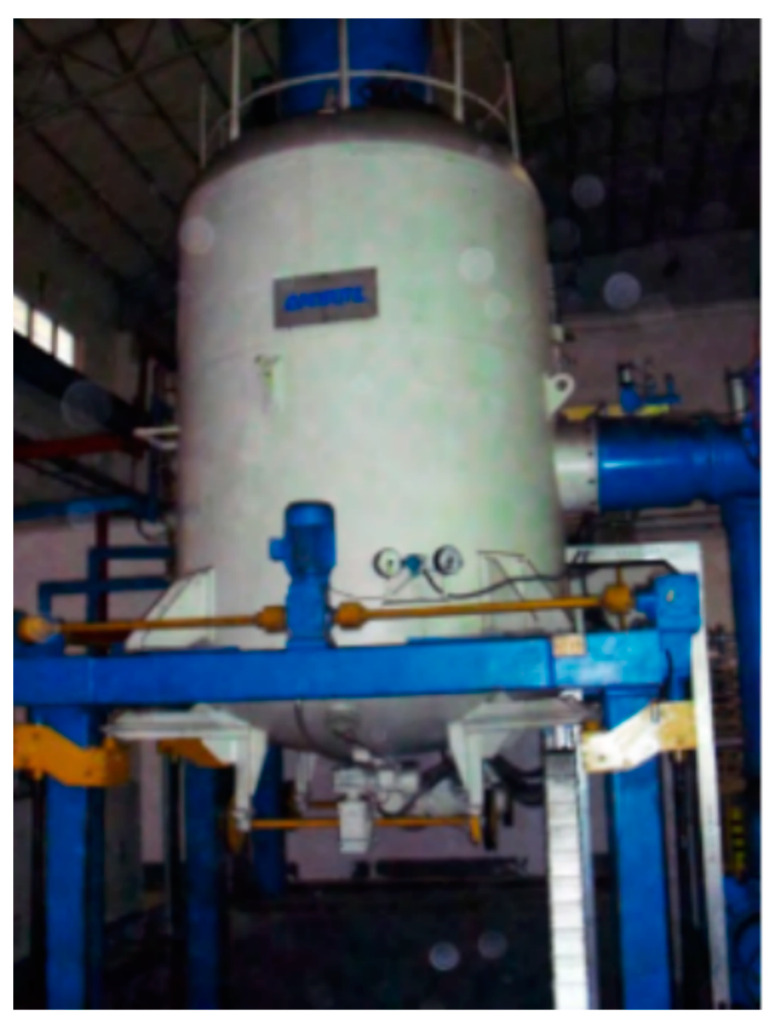
Vertical vacuum gas quenching furnace [22].

Horizontal double-chamber gas quenching vacuum furnace mainly consists of two parts: a heating chamber and an inlet and outlet chamber, both of which are equipped with lifting heat insulation [20]. The furnace body, with a double-layer structure, facilitates the circulation of cooling water through the middle region. An automatic transfer mechanism is integrated into the furnace body, allowing the material frame to move back and forth between the two chambers as per a specified procedure. This internal-heat-type vacuum heating equipment features high thermal efficiency and a large furnace loading capacity. It is especially suitable for the processing of large workpieces and can realize rapid heating/cooling and high reliability in the use process. Moreover, it shortens the workpiece heat treatment cycle and reduces production costs. The precise automatic control system configuration realizes automatic programming and processes random change, ensuring automatic heating in the vacuum state, automatic workpiece transfer, and quenching, which improves the heat treatment process quality and the part’s metallographic structure. The vertical vacuum gas quenching furnace is vacuum heat treatment equipment designed for long workpieces that need to be treated upright. It comprises an equipment body, lifting mechanism, water cooling system, inflation system, vacuum unit, pneumatic system, electrical system, and hydraulic system, as depicted in Figure 1. A high-power motor with a gas circulating fan and a heat exchanger are mounted on the side of the furnace. Cooling gas flows through the gas loop from the hot zone to the gas cooler and then into the hot zone from the top or bottom, while the air flow can be circulated in reverse to quench the workpiece to be treated. This cooling method reduces the temperature difference of the workpiece in the cooling process and effectively prevents the distortion of the workpiece.

A dual-vacuum furnace with the heater in the outside vacuum has been developed at the Institute of High Energy Physics (IHEP) to perform nitrogen doping (N-doping) heat treatment of 1.3 GHz 1-cell niobium cavity [21]. N-doping can reduce the cost of the cryogenic system greatly. In China, Shanghai High repetition rate XFEL and Extreme light facility (SHINE) began construction in 2018, which requires 600 1.3 GHz 9-cell cavities with high *Q*_0_. The performance of the furnace is key to the success of the N-doping. Therefore, a new dual-vacuum furnace was developed at IHEP to obtain higher performance. This furnace is equipped with a stainless steel inner chamber, and the heater is in the outer vacuum chamber. So, the inner vacuum chamber is much cleaner with a better vacuum, which is beneficial for N-doping. A lot of N-doping experiments have been carried out with this furnace, and the good results have already been observed.

### 2.3. Development of Vacuum High-Pressure Gas Quenching Technology

Heating in a vacuum is entirely dependent on radiant heat transfer, which is especially slow for dense loads consisting of a large number of workpieces. Wang studied the influence of vacuum heat treatment heating lag time on the hardness and microstructure of 4Cr5MoSiV1 die materials by combining numerical simulation and process experiment [23]. They pointed out that the heating and holding time of vacuum heat treatment calculated by empirical formula is too long, which is not conducive to energy saving and emission reduction [23]. If the gas can be circulated through the workpieces during the heating phase, convective heat transfer will significantly increase the heating rate and shorten the heating time. Convective heating technology refers to the vacuum furnace being pumped to a certain vacuum degree, and then through 0.1–0.2 MPa inert (Ar, He), reducing N_2_ or reducing H_2_ gas, and heating under the condition of full agitation. Compared to a pure vacuum condition, the heating speed can be at least doubled by using the convective heat technology.

For this, vacuum furnaces with dual heating units were developed. This type of vacuum furnace includes a convective heating system and a pure radiant heating system, both of which simultaneously heat the workpiece to the final austenitizing temperature, greatly reducing the heating time. 

In the production practice, the low heat transfer efficiency in gas quenching furnaces remains a persistent challenge. The performance of heat exchangers directly determines whether the gas quenching furnace can meet the requirements of high efficiency, energy saving, and being clean and pollution-free. To improve the cooling rate of vacuum gas quenching, the heat exchangers can be improved to reduce the quenching gas temperature.

**Figure 2:**
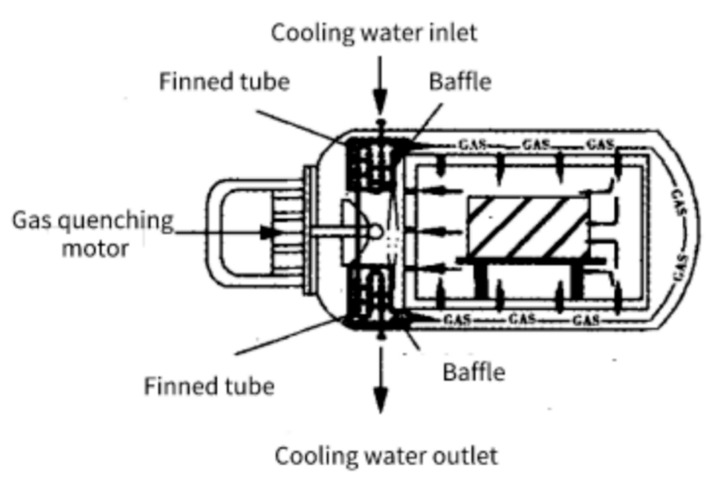
Heat transfer structure of vacuum gas quenching furnace [24].

Wei Lei et al. [24] improved the structure of conventional heat exchangers in certain high-pressure vacuum gas quenching furnaces by changing the heat exchange optical tube into a fin tube, changing the baffle from bow to spiral, and replacing the steel heat exchange material with copper. Compared with the traditional heat exchange equipment, the improved structure achieved the effect of heat transfer enhancement and effectively improved the heat transfer capacity of the heat exchange equipment, as shown in Figure 2.

In order to obtain the best quenching results in terms of microstructure, hardness, and distortion, it is essential to set reasonable gas quenching parameters, including austenitizing temperature, isothermal temperature, gas quenching pressure, isothermal time and holding time. 

**Figure 3:**
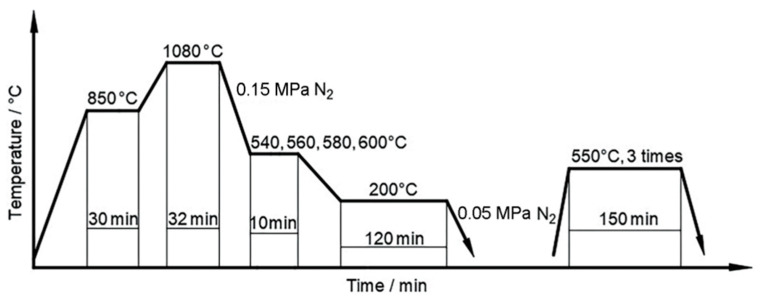
Heat treatment regime involving vacuum graded quenching and tempering process of 8Cr4Mo4V steel [26].

With the rapid advancement of AC speed regulation technology since the 1980s, frequency conversion technology has been significantly developed and gradually implemented in the actual production. The gas quenching chamber adopted frequency conversion technology on the gas quenching stirring fan, which could make the quenching cooling process and CCT curve as close as possible [25]. Moreover, the distortion control of quenched parts has become more scientific and flexible, in line with the flexible production concept that is widely embraced by the manufacturing industry. This approach has provided a strong guarantee for the realization of the gas fractional quenching process. Compared with ordinary gas quenching processes, the hierarchical gas quenching process is more flexible and effective in controlling quenching distortion. Yu et al. [26] carried out graded quenching of 8Cr4Mo4V steel for aviation bearings at different temperatures under vacuum conditions. The vacuum step quenching and tempering process is shown in Figure 3. Vacuum-graded quenching can improve the comprehensive mechanical properties of 8Cr4Mo4V steel. Compared with ungraded vacuum quenching, the impact toughness of the steel is increased by 23.3%, and the rotating bending fatigue limit is increased by 110 MPa. 

The dynamic quenching process is divided into three steps: (1) High-strength quenching until the temperature of a certain part is reached; (2) reducing the quenching strength for a set period to balance the part’s temperature; and (3) increasing the quenching strength again until the end of the quenching process. This process has been successfully applied to the heat treatment of transmission gears. After heat treatment, the distortion of gears is kept at an extremely low level and no subsequent processing is required, thus saving costs for transmission manufacturers [27,28].

Yu et al. [29] introduced a novel dynamic quenching–partitioning process for large-scale products utilizing water spraying, taking into account critical bainite transformation kinetics within this process. This study explores the feasibility of implementing the Quenching and Partitioning (Q&P) process through spraying, which involves an initial quenching step from full austenitization to a quench-stop temperature (Tq) below the martensite start temperature (Ms). Subsequently, a dynamic isothermal holding stage is applied at a temperature above Tq during the partitioning step, followed by the final quenching phase. To facilitate efficient cooling, an air–water alternate quenching strategy was employed, spanning time intervals of 0–35 s and 60–300 s. The total computational duration for the entire process was set at 3600 s, with an initial calculated part temperature of 900 °C following austenitization. The application of the Quenching and Partitioning (Q&P) process to large-scale components offers notable advantages, encompassing energy savings and cost-efficiency while maintaining essential material properties. This novel approach presents a promising avenue for the production of large-scale components.

## 3. Experimental Study of Vacuum High-Pressure Gas Quenching and Main Influencing Factors

### 3.1. Comparison of Vacuum Gas Quenching and Other Quenching Methods

Vacuum oil quenching and vacuum gas quenching are two commonly used vacuum quenching processes. The latter utilizes N_2_ and other gases as cooling media. Compared to oil, using gas as the quenching medium can reduce the distortion of the workpiece due to the single-phase nature of gas convection cooling, thereby avoiding state changes during the process. Compared with workpiece quenched under atmospheric pressure (salt bath furnace, well furnace, mesh furnace, box furnace, controlled atmosphere furnace, etc.), the hardness of a vacuum gas quenching workpiece is more uniform and slightly higher, mainly because the workpiece surface is active during vacuum heating, without decarbonization, oxide film hindering cooling, etc. For a HSS workpiece with a diameter of 50 mm, the hardness after vacuum gas quenching is HRC64, but when the diameter is greater than 100 mm, the hardness of the workpiece is HRC60, which is lower than that of salt bath quenching. The hardness of die steel Cr12MoV after vacuum gas quenching is HRC59-61, and the hardness after oil quenching is HRC60-63, among which the hardness of a workpiece larger than 100 mm after gas quenching is below HRC60, and it is in the incomplete quenching state. The 200 mm forging die steel SKT4 oil quenching (heating temperature of 800 °C), which reaches hardness up to HRC58, can meet the requirements of the use of a die [30].

Lin et al. [31] conducted comparative studies involving oil quenching and high-pressure vacuum gas quenching techniques on Cr12MoV material. It was found that after two different heat treatments, the heat-treated steel possessed a structure comparing martensite, carbide, and residual austenite, with a significant improvement in hardness. Among them, the surface of the sample treated by vacuum gas quenching was clean and neat, and there was no need to clean the surface oil in the subsequent treatment, and it was very friendly to the environment.

Yu et al. [32] found that the grain growth rate of HSS was not obvious under vacuum heating. In the quenching temperature range, the grain size of W6Mo5Cr4V2 steel changes in the range of 11 grades with the difference of pre-hot rolling deformation. After comparison, there is basically no difference compared with that after salt bath quenching. However, at quenching temperatures exceeding 1220 °C and heating and holding coefficients beyond 40 s/mm, grain growth is noticeable with prolonged holding time. When the quenching temperature is 1220 °C and the insulation coefficient is >60 s/mm, or the quenching temperature is ≥1230 °C and the insulation coefficient is ≥30 s/mm, abnormal grain growth occurs in the vacuum gas quenching with a workpiece size of φ20 × 20 mm. Many fine carbide particles exist in the form of chains in samples that produce mixed crystals. The size of the particles varies greatly (as small as 0.1 μm and as large as 3.5 μm), and the distribution is uneven. 

**Figure 4:**
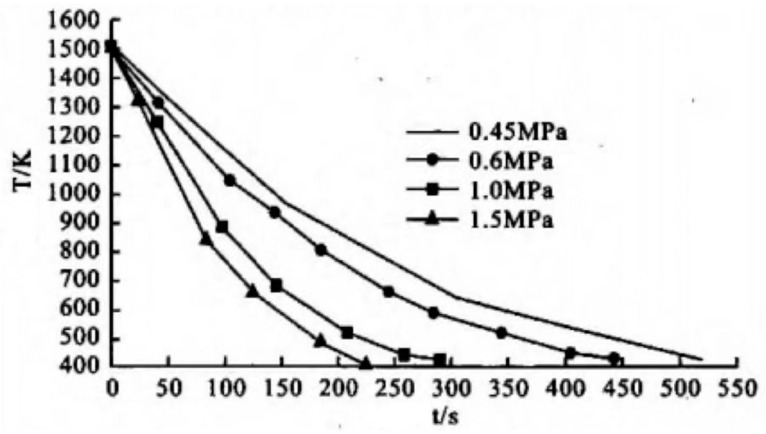
Effect of quenching gas pressure on the workpiece cooling rate [32].

**Figure 5:**
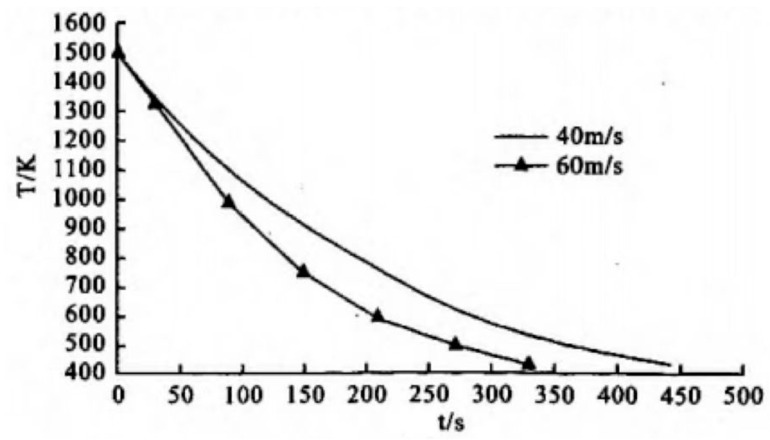
Effect of quenching gas velocity on workpiece cooling rate through computer simulation [32].

In the quenching temperature range of 1180~1240 °C, W6Mo5Cr4V2 steel exhibits high hardness after vacuum gas quenching. In addition, the hardness value after quenching gradually increases with an increase in quenching temperature, reaching a peak at 1220 °C. When quenching is conducted above 1220 °C, the quenching hardness decreases due to the increase in the residual austenite volume in the steel. The hardness of HSS after gas quenching and tempering also increases with the increase in the quenching temperature. After true air quenching at 1220 °C and salt bath tempering at 540 °C three times an hour, the hardness of HSS can reach HRC 64–65. However, when compared to the hardness of salt bath quenching and tempering under the same conditions, the hardness values of vacuum gas quenched and tempered steel are lower. The low tempering hardness and red hardness of vacuum gas quenched HSS are related to the low cooling rate during vacuum gas quenching. As shown in Figure 4 and Figure 5, it is well-known that the cooling rate increases with the gas pressure. In order to avoid the precipitation of secondary carbides and achieve high hardness of HSS tools with large section sizes obtained after vacuum gas quenching and tempering, vacuum gas quenching can be adopted [32].

Wang et al. [33] studied the properties of 1Crl7Ni2 alloy steel after vacuum oil quenching and vacuum gas quenching, respectively, and compared their microstructures. After oil quenching and gas quenching with the same process parameters, the mechanical properties of 1Crl7Ni2 steel were improved. Increasing the quenching pressure during gas quenching led to an increase in the tensile strength of 1Crl7Ni2 steel to some extent.

Yan et al. [34] conducted vacuum quenching, tempering, and secondary tempering of 4Cr5MoSiV1 steel for die casting at different temperatures, and analyzed the microstructures of 4Cr5MoSiV1. It was indicated that 4Cr5MoSiV1 presented excellent mechanical properties after vacuum quenching at 1050 °C and twice tempering at 600 °C. The heat treatment procedures of the die casting materials commonly used in the world, especially 4Cr5MoSiV1, were described and compared by using various processes such as air quenching, tempering and isothermal spheroidizing annealing [35,36,37].

Syifa Luthfiyah et al. [38] using 4Cr5MoSiV1 steel subjected to vacuum heat treatment at 1030 °C for 3 h and progressively quenched at different quenching rates. The results showed that the hardness, structure, and corrosion resistance of the material surface are affected by the quenching speed. The difference in the cooling speed of each sample resulted in variations in surface properties. The 4Cr5MoSiV1 steel corrosion resistance was determined in the entire vacuum heat treatment process since the final phase formed and air condition will be the determining factors of how resistant the material is to corrosion.

R. Sola et al. [39] studied the effects of laser, vacuum, and induction hardening on the mechanical properties, wear resistance, and corrosion resistance of stainless steel AISI420. The results showed that all three heat treatments greatly improved the wear resistance of the steel due to the increase in hardness. Laser and vacuum quenching also increased the corrosion resistance, while the corrosion resistance after induction quenching is lower than that of untreated steel. The effect of different kinds of quenching, i.e., laser, vacuum, and induction ones, on the mechanical properties and wear and corrosion resistance of stainless steel AISI 420 was studied. It was shown that all three kinds of heat treatment considerably increased the wear resistance of the steel due to growth in the hardness. Laser and vacuum quenching also increased corrosion resistance. After induction quenching the corrosion resistance was lower than in untreated steel.

In summary, vacuum gas quenching achieved no oxide skin on the surface, and the distortion degree was small. The microstructure was more uniform. For parts with low precision requirements, there can be reduced or no subsequent processing, and successful experiments have been conducted on different steel grades, and significant results have been obtained, as shown in Table 2. 

Vacuum heating prevents particle oxidation through degassing, ensuring excellent surface quality without the need for post-quenching treatment. This process is environmentally friendly as it avoids the use of harmful gases, while steel subjected to vacuum quenching exhibits enhanced mechanical properties. Vacuum quenching can greatly reduce the hydrogen content and other gas content of high-strength steel prone to hydrogen embrittlement so that the ability of steel to resist brittle fracture has been improved. At the same time, the service life after vacuum quenching is longer than that of conventional heat treatment. In the heat treatment of different kinds of steel, the vacuum gas quenching process is very advantageous, and vacuum heat treatment is also the most appropriate heat treatment method at present, so it is worth focusing on the development [40].

### 3.2. Essential Factors Affecting Vacuum High-Pressure Gas Quenching

The process of gas quenching is resulting in a complex flow path that perturbs the flow during cooling and induces a complex heat transfer field. Thuvander et al. [41] studied the gas-quenching process and showed how heat transfer homogeneity influences the uniformity of the mechanical properties, in particular the hardness, of gas-quenched metal parts. Two different factors can contribute to the lack of heat transfer uniformity in a gas-quenching furnace. Firstly, the design of the furnace must ensure a uniform distribution of flow around the charge. The design of the furnace usually reflects the consideration of a large number of factors such as cost, weight, and size. Strong optimization concerning other factors can sometimes result in complex flow paths giving rise to strong variations in the gas velocity. Secondly, as most bodies to quench are bluff bodies, the occurrence of flow separations results in a local variation of heat transfer properties [42,43,44,45].

**Figure 6:**
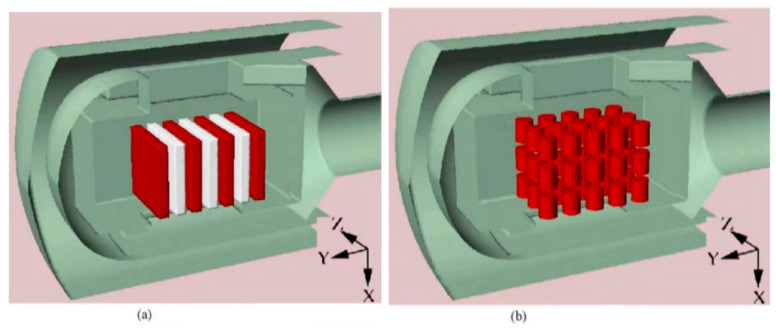
Coordinates system used within the quenching chamber: (**a**) case with plates and (**b**) case with cylinders [40].

**Figure 7:**
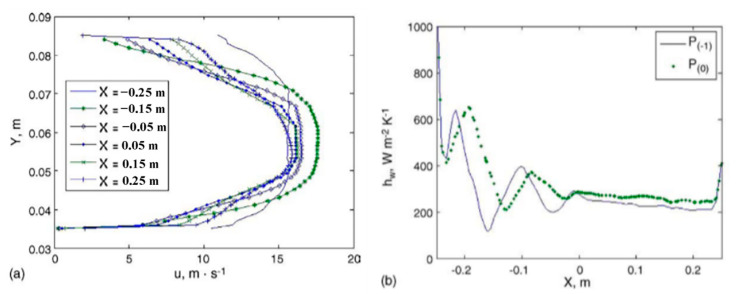
Relation between velocity profile and heat transfer coefficient evolution in the uniform flow region—case with plates. (**a**) Velocity profiles between plates *P*_(0)_ and *P*_(−1)_. (**b**) Heat transfer coefficient distribution on plates *P*_(0)_ and *P*_(−1)_ [40].

The temperature uniformity and system accuracy of heat treatment equipment are the core indexes that reflect the processing performance of heat treatment equipment, and ultimately affect the quality of heat treatment parts. Macchion et al. [39] investigated the relationship between flow patterns and heat transfer within the charge placed in the furnace. As shown in Figure 6 and Figure 7, the interaction between flow behavior and heat transfer is of crucial importance for the efficiency of the gas quenching process. Bless et al. [46] and Pritchard et al. [47] performed studies of gas quenching processes within gas quenching furnaces, but did not address the relationship between design, flow pattern, and heat transfer within the working chamber. In their work, thermal effects in a gas quenching furnace were investigated using numerical simulations. Computation of mean heat transfer rates over the charge indicates that the heat transfer rates substantially lower (by 20–50%) than the metal pieces in the middle of the charge, depending on the type of load and position. The standard deviation of the heat transfer coefficient is about the same magnitude in the case with cylinders as in the case with plates, with a slightly larger non-homogeneity in the case with cylinders. As a result, the heat transfer non-uniformity is the interaction of three geometrical parameters: (1) geometry of the quenching chamber, (2) charge configuration, and (3) load parts shape. As such, it would seem reasonable to propose equipment either guiding equipment within the charge to limit the influence of neighboring bodies or only the central area of the quenching chamber for quenching. 

## 4. Current Situation of Computer Simulation of Vacuum High-Pressure Gas Quenching Process

**Figure 8:**
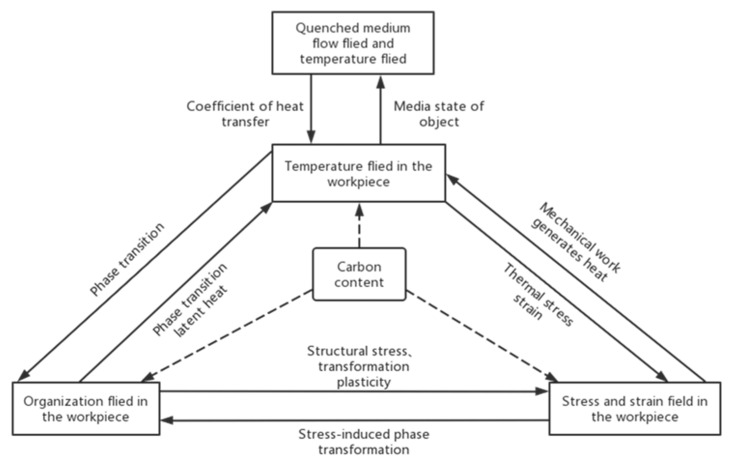
Coupling of the quenching process [48].

The temperature field, stress field and phase transition in heat treatment are interrelated and inseparable. The quenching process involves the instantaneous change and interaction of temperature, phase transformation, stress, strain and medium flow direction, which is a very complicated process. Some scholars have established a coupling diagram according to the relationship between the fields, as shown in Figure 8 [48]. During the quenching process, the effect of the temperature field on phase transformation is manifested in that austenite decomposes with temperature change, resulting in phase transformation. In the process of phase transformation, the latent heat of phase transformation is generated to form an internal heat source, which will affect the temperature field. Due to the uneven cooling rate in each region during the cooling process, the thermal stress is generated. The stress field distribution is affected by the phase transition stress and superplasticity at different positions between the new phase and the parent phase. The internal stress changes the rate of phase transition (phase transition dynamics). The transition from inelastic energy to heat energy occurs between microstructures due to stress, but this part of the energy is generally not considered because of its small influence.

The adoption of numerical simulation technology stands to enable a shift in the heat treatment industry, from a reliance on traditional experience-based approaches towards computer-based predictive modelling. The use of computer modeling and simulation is conducive to the design of engineering materials and the prediction of the properties of these materials, while greatly reducing both time and cost expenditures [49,50,51,52,53]. 

Numerical simulation of the flow process is a numerical analysis process by solving continuum equations, motion equations, and energy equations. The numerical simulation of turbulent motion can be divided into three types: direct simulation (DNs), large eddy simulation (LES) and Raynaud time mean equation simulation (RAES). The difference between the three models lies in that the turbulence kinetic energy and dissipation rate transport equation of the standard model is derived under the condition that the convection and diffusion effects are considered, but neglect the effects of the near-wall viscosity. The renormalization group (RNG) model derives the same equations, but with coefficients derived from the renormalization group theory, leading to better generality. The Realizable model proposed by Shih et al. [54] differs from the standard model in that the turbulence dissipation rate equation source term (turbulence coefficient) is no longer a constant, but a function determined by the vortex tensor. Comparative studies between numerical simulations and experimental results for turbulence and temperature fields have shown that (1) a suitable turbulence model and boundary conditions can yield accurate results for vacuum gas quenching; and (2) the three models are applicable to different fields due to their different treatment of the turbulence dissipation rate. The Realizable model, which has a strong anisotropy, agrees well with reality, while the standard model shows significant discrepancies.

### 4.1. Influence of Structure of Gas Quenching Furnace on Gas Quenching

Currently, the simulation of vacuum gas quenching mainly focuses on the calculation of temperature field, flow field, cooling rate, workpiece cooling curve, phase transition, and workpiece performance under different conditions, as well as the analysis of furnace strength and structure. Jin et al. [55] studied the safety of the horizontal single-chamber vacuum gas quenching furnace, wherein the structural composition of the furnace shell, furnace door, and gear ring were identified as the main components that affect equipment safety. The furnace shell and gear ring are calculated theoretically by the calculation and charting methods, and the results showed that the stress distribution of the furnace shell basically met the requirements of material strength, and the stress in the root region of the gear ring exceeded the allowable stress and produced a large stress concentration value.

Wang et al. [56] studied the FGF-644H pipeline-type and VHQ-7712 press-in vacuum gas quenching furnaces by conducting numerical simulations and physical verification experiments on the gas quenching process using 4Cr5MoSiV1 die steel as the specimen. The research results showed that the cooling rate of the pipeline vacuum gas quenching furnace was faster than that of the pressed vacuum gas quenching furnace under the same cooling pressure. Additionally, the cooling rate of the furnace door was observed to be faster than that of the furnace bottom, and the pipe vacuum gas quenching furnace was found to be more suitable for quenching small workpieces.

Monesh et al. [57] studied the influence of quenching pressure, the distance between the quenching probe, and the tip of the nozzle (SOD) on the quenching process. The inverse heat conduction (IHC) method was used to calculate the surface heat flux. The results showed that an increase in pressure led to an increase in the cooling rate and peak surface heat flux, while an increase in nozzle spacing led to a decrease in the surface heat flux and cooling rate. The effect of increasing SOD by 1 cm was greater than that of decreasing the gas pressure by 0.2 MPa. Wang et al. [58] numerically simulated the gas–solid coupling flow heat transfer process in a nozzle-type vacuum gas quenching furnace containing a workpiece. They used a response surface method to establish a fitting model and optimized the structure of the air duct. In the study of Zhe et al. [59], compressed air as the quenchant at different pressures of 0.4 MPa, 0.6 MPa and 0.8 MPa and different SOD of 9 cm, 10 cm and 11 cm were tested to study the effect of these two parameters on quenching heat transfer rates. Surface heat flux computations were carried out using the IHC method, with the measured time-temperature data as input. They found that an increase in pressure led to an increase in the cooling rate and peak surface heat flux, while an increase in nozzle standoff distance resulted in a drop-in cooling rate and peak surface heat flux.

### 4.2. Simulation of Workpiece Temperature Field and Phase Transition

The calculation of the temperature coefficient significantly impacts the analysis of thermal stress and strain during quenching, as well as the residual stress and microstructure of the workpiece after quenching. Yuan et al. [60] used the finite element analysis software DEFORM V11 to establish a numerical analysis model, then simulated the temperature field changes, microstructure field changes, and distortion of the parts during vacuum quenching. By comparing predicted results with experimental data, the authors concluded that the inverse heat transfer method provided a highly reliable heat transfer coefficient, and the finite element analysis model accurately reproduced the heat treatment process of large aircraft thin-walled parts. Sugianto et al. [61] used DEFORM 2D and 3D and developed a finite-element model to study phase transformation kinetics during the heating, carburizing, diffusing, and quenching processes. 

Cheng et al. [62] pointed out that heat conduction during quenching was a nonlinear problem, and the accurate calculation of temperature was significantly reliant on the surface heat transfer coefficient and phase transformation.

Cheng et al. [63] simulated the CCT diagram of 42CrMo steel by mathematical transformation and calculated the volume fraction of phases based on the TTT diagram. The thermophysical properties were viewed as a function of temperature and the volume fraction of the phase composition. Finally, the finite element method was used to calculate the temperature field with phase transformation and nonlinear surface heat transfer coefficient, with the corresponding temperature function established. In the finite element method-based temperature calculations during quenching, the use of smaller time steps can sometimes introduce unwanted “oscillations” in numerical solutions. These oscillations have the potential to significantly affect the accuracy of temperature, thermal stress, and residual stress predictions. To mitigate this issue, a rational approximation method was employed for finite element analysis to calculate the temperature field during quenching. The results showed that this method can effectively avoid the “oscillation” of numerical solutions with small time steps, and was not limited by the element form with high precision. The CCT diagram of the 42CrMo steel was simulated by mathematical transformation, and the volume fraction of phase constituents was calculated. The thermal physical properties were treated as functions of temperature and the volume fraction of phase constituents.

Hao et al. [64] established a three-dimensional numerical model for simulating the transient heat transfer in a vacuum heat treatment furnace that incorporates a PID temperature control module. They used this model to conduct numerical simulation and analysis on the vacuum heating process of a rod-shaped workpiece in vacuum heat treatment furnaces, heat insulation layer of vacuum heat treatment furnace, and heating tube, leading to significant improvements in the heating process. The findings revealed that for small workpieces placed in order, a rapid heating process could be employed without causing any thermal distortion of the workpiece, thereby reducing the heating time and cost.

### 4.3. Simulation of the Flow Field in Vacuum Furnace

Schmidt [65] utilized a multi-scale model to simulate the flow in a commercial gas quenching chamber to provide a faster rate of convergence. Finally, a cylindrical part was used for quenching in a two-chamber vacuum furnace to verify the model results. The efficacy of various upstream velocity distributions was demonstrated with a cylindrical part quenched in a two-chamber vacuum furnace. The multi-scale simulation method and flow process studies yielded promising results. 

Lior et al. [66] simulated the flow of cooling gas in a complex quenching chamber to investigate the underlying causes of the uneven flow. The research showed that the uneven flow rate was mainly caused by the flow mode determined by the chamber design and was independent of the gas type. Luo et al. [67] numerically simulated the process of workpiece gas quenching by using the computational fluid dynamics (CFD) method and established a three-dimensional unsteady model of a vacuum gas quenching furnace on a FLUENT platform. The distribution of the flow field and workpiece temperature field in the furnace, the influence of gas type, gas pressure, and speed on the cooling rate of cylindrical simplex during the quenching process were simulated, and the quenching cooling process of a multi-workpiece was simulated. Cooling curves of workpieces at different positions in the furnace were predicted. The simulation results were in good agreement with the experimental results, which provided a theoretical basis for the optimization of the gas quenching process. Schmidt et al. [65] explored the influence of workpiece type and arrangement on the uniformity of gas quenching, and calculated the flow conditions and the corresponding heat transfer rate distribution of different furnace loading modes. They demonstrated that the pressure equilibrium in the plane perpendicular to the main flow direction could yield a more uniform flow distribution, and that workpieces should be placed closer together in the low-velocity charging area. When the cylinders were not aligned neatly, the fluidic flow structure favored better heat transfer for the bottom workpiece.

### 4.4. Simulation of Mechanical Properties of Workpieces

The performance of the workpiece after heat treatment is closely related to its shape, position in the furnace, the state of the cooling medium, and the flow direction during the cooling. Accurate prediction of the mechanical properties of quenched steel parts has been the focus of recent research efforts. The results of the quenching factor analysis (QFA) method were applied to predict the hardness of some quenched parts [68,69], but its accuracy decreased with the decrease in hardness. Sajjadi et al. [70] modified the QFA method to predict the hardness of CK60 steel after austenitizing and quenching at 830 °C, and the results showed that the accuracy was significantly improved. Kianezhad et al. [71] utilized the neural network method (ANN) to predict the properties of quenched steel in one step. While the QFA method is limited to accurately predicting the hardness of the main phase (martensite) in steel, the ANN can predict the properties of different phases in quenched steel workpieces.

During the gas-quenching process, the heat-transfer coefficient plays a critical role in determining the final product properties, such as hardness, strength, residual stress and distortion. Li et al. [2] optimized the heat transfer coefficient by response surface method to improve the mechanical properties of the workpiece, and used the finite element package DEFORM to predict the material responses during the quenching process. The results showed that numerical experiments significantly reduce the experimental costs in optimizing or improving gas-quenching processes. Zhang et al. [72] investigated the evolution of stress and distortion in three different quenching processes, including water quenching, oil quenching, and vacuum gas quenching for U-shaped AF1410 steel samples, using experimental and numerical methods. They developed a thermo-metallurgical–mechanical coupled model to predict the magnitude and distribution of phase fraction, stress, and distortion accurately. The Abaqus user subroutines UMAT was utilized to predict stress and distortion, and the mechanical properties of the mixed phase were calculated by the linear mix rule according to the phase fraction to consider the influences of solid-state phase transformation (SSPT), including the transformation strain and transformation plasticity. The results indicated that the distortion of the U-shaped sample quenched by water, oil, and vacuum gas was decreased sequentially.

## 5. Summary

Non-oxidation heating represents a prominent trend in the advancement of modern heat treatment technology, with vacuum gas quenching technology and equipment standing out as one of its primary manifestations [73]. Vacuum gas quenching offers numerous advantages, including oxidation-free, decarburization-free, pollution-free, and bright surface finishes, enabling easy automatic control and elimination of post-quenching cleaning procedures. Furthermore, it reduces distortion during treatment, enhances the surface quality of the workpiece, and facilitates continuous automatic production, holding a wide development prospect. Currently, gas cooling pressure in advanced gas quenching furnaces has reached 8 MPa, 10 MPa, or even more than 15 MPa, with vacuum gas quenching furnaces being manufactured to meet the microstructure and performance requirements of certain material heat treatment. Concurrently, computer modeling is emerging as an invaluable tool, with progress in materials engineering closely intertwined with computational methods’ application and development. Computer-aided processes and phenomena reproduction permit accurate property prediction and significantly curtail financial expenditure and experimentation time. Future improvements in vacuum gas quenching may be pursued from the following directions:

(1) The vacuum gas quenching furnace with high thermal conductivity, low cost, high cooling rate and good safety are expected to be developed to ensure the safe use of hydrogen or mixed gas quenching medium, so as to meet the high efficiency gas quenching requirements of large and complex workpieces.
(2) Precise control of heating and cooling speed to obtain the best microstructure and properties of materials. This can be attained through the integration of an automatic adjustment damper into the cooling path, along with temperature and airflow sensors, all of which are incorporated into a sophisticated control system. With these advanced features, the heating and cooling speed can be precisely calibrated to ensure the desired outcome, allowing for unparalleled control over the material’s microstructure and properties.
(3) Determining the critical diameter of vacuum gas quenching of different steels under different quenching pressures and loading capacities is convenient for process production.
(4) Developing high-precision simulation software for vacuum gas quenching based on specific furnace types can help to meet the requirements of personalized optimal vacuum gas quenching processes with different materials and different loads.
(5) Developing the special furnace for vacuum gas quenching and heat treatment of extremely widely used parts and its auxiliary software system can help to realize the standardized and automatic production of the best effect and the least energy of vacuum gas quenching and heat treatment of parts.

## Figures and Tables

**Table 1:** The development of global high-pressure gas quenching furnace.

Time	Development Circumstance	Characteristics
1975	The world’s first vacuum high-pressure gas quenching furnace	Its cooling rate was higher and more uniform than previous gas quenching furnaces.
1980	NVFC series high-pressure vacuum heat treatment furnace	Quenching at 1.7 atmospheres had enough cooling speed to enable workpieces to meet the hardness requirements.
1981	Introduced and installed one ZC-30 and one ZC-65 vacuum quenching furnace and one ZCT-65 vacuum carburizing furnace.	Increased the workpiece life by more than one time, and even tens of times, compared with salt bath heat treatment.
1983	China’s first pressurized vacuum gas quenching furnace	Improving the cooling capacity of the domestic vacuum gas quenching furnace.
1988	0.5 MPa gas quenching vacuum furnace (VDN-513R)	a single-chamber horizontal internal circulation cooling gas quenching furnace, and the gas cooling pressure could be selected within the range of 0.2 MPa to 0.5 MPa.
	China’s first high-pressure vacuum gas quenching furnace	Capable of being pressurized to 0.5 MPa.
The end of the 20th century	Vacuum gas quenching furnaces of 0.2, 0.6, 0.8, 1, and 2 MPa	With vertical and horizontal types.
	Two-chamber vacuum furnaces called DualTherm	Compared to single-chamber vacuum furnaces, the double-chamber vacuum furnace has better cooling capacity, and the cooling effect of the 0.2 MPa N_2_-cooled double chamber furnace is equivalent to that of the 0.4 MPa single chamber furnace.
	5 MPa gas quenching vacuum furnaces	Not only share the advantages of ordinary vacuum gas furnaces (good surface brightness of heat-treated products, small distortion, and no secondary cleaning), but also greatly expand the range of materials and section size of treated workpieces.
2001	A modular system of the ModulTherm	The system is extremely flexible. Each processing room can be operated independently, allowing individual processing rooms requiring maintenance to be closed separately, while the other processing rooms operate normally.
2013	HZQL-450 vertical gas quenching vacuum furnace with 1 MPa high-pressure and high flow rate	Increase the loading capacity to 2500 kg.

**Table 2:** Steels successfully subjected to vacuum heat treatment.

Steel Grade	Heat Treatment Method	Achieving Results
30CrMnSiNi2A	Vacuum quenching	No oxidation or decarburization on the surface.
8Cr4Mo4V	Vacuum step quenching and tempering process	Toughness is increased by 23.3%, and the rotating bending fatigue limit is increased by 110 MPa.
Cr12MoV	Vacuum gas quenching	A significant improvement in hardness, the surface was clean and neat.
W6Mo5Cr4V2		The hardness value increases with an increase in quenching temperature, reaching a peak at 1220 °C.
4Cr5MoSiV1		Excellent mechanical properties, the hardness, structure, and corrosion resistance of the material surface are affected by the quenching speed.
1Crl7Ni2	Oil quenching and gas quenching	Gas quenching led to an increase in the tensile strength of 1Crl7Ni2 steel.
AISI420	Laser, vacuum, and induction hardening	Vacuum quenching improved both wear resistance and corrosion resistance.

## Data Availability

No new data were created or analyzed in this study. Data sharing is not applicable to this article.

## References

[1] Wu S.Q. (2002). Vacuum High Pressure Gas Quench Heat Treatment. Die Mould Manuf..

[2] Li Z., Grandhi R.V., Shivpuri R. (2002). Optimum design of the heat-transfer coefficient during gas quenching using the response surface method. Int. J. Mach. Tools Manuf..

[3] Wang Z.J., Xu C.H., Li F.Z., Wang B.X., Li M.C., Hui Z. (2002). Development in technique and equipment of high-pressure gas quenching vacuum furnaces. Vacuum.

[4] Zhu Y.Y. (1992). High pressure gas quenching vacuum heat treatment process and equipment at home and abroad. Eng. Des. Appl. Res..

[5] Zhou H., Wu N., Ma L. (2009). Application of Heat Treatment Vacuum Quenching Process. Hongdu Sci. Technol..

[6] Samuel A., Prabhu K.N. (2022). Residual Stress and Distortion during Quench Hardening of Steels: A Review. J. Mater. Eng. Perform..

[7] Yu L., Yang Y. Application status and development direction of vacuum high-pressure gas quenching technology in China. Proceedings of the 19th Heat Treatment Technology Exchange Conference in North China and the 12th Annual Academic Meeting of Tianjin Heat Treatment Society.

[8] Pan Z., Zhu Y.X., Bian K.Q. (2019). Exploration of vacuum gas quenching process. Met. Work..

[9] Shen Z.X. (1984). Vacuum air quenching. Hot Work. Technol..

[10] Zhou Z.H. (1988). Practical application of vacuum heat treatment in 20 cases. Heat Treat. Met..

[11] Gong X.N., Liu Q., Liu Z.G., Bai Y.S. (1990). HPV-200 high-pressure vacuum air quenching furnace equipment and process. Vacuum.

[12] Zhang H.K., Chen S.M. (1993). Polish high-pressure gas quenching vacuum furnace and domestic auxiliary facilities. Heat Treat. Met..

[13] Fan D.L., Tian R.Z., Kowalewski J. (1999). New Progress of Vacuum Heat Treatment Technology—High and Superhigh Pressure Gas Quenching. Heat Treat. Met..

[14] Heuer V., Loser K., Busch G. Professional heat treatment service for automobile industry. Proceedings of the 6th China Heat Treatment Week.

[15] Zhang H.K. (1996). Progress and Application of Vacuum Air Quenching Furnace. Hot Work. Technol..

[16] Gao W., Gao F.L., Yang J.L. (1997). Development of a new type of high-pressure vacuum air quenching furnace. Mech. Des. Manuf..

[17] Song J.L., Meng L., Liu X.W. Research and Development of HZQL-450 Vertical High Pressure Gas Quenching Vacuum Furnace. Proceedings of the 50th Anniversary Conference of the National Society for Heat Treatment and the 9th China Heat Treatment Week.

[18] Zhang J.F., Jin L.Y., Cheng J.P., Yang R.H. (2009). Development of gas quenching vacuum furnace. Shanxi. Sci. Tech..

[19] Hou W.Q., Zhang J.F. (2008). Development of Gas Quenching Vacuum Furnace. Electron. Process Technol..

[20] Ma W.D. Development of a new horizontal double-chamber vacuum air-cooled oil quenching furnace. Proceedings of the 7th Quality Work Conference of the Director and Manager Meeting of China Heat Treatment Industry.

[21] Liu B.Q. (2021). Nitrogen doping with dual-vacuum furnace at IHEP. Nucl. Inst. Methods Phys. Res. A.

[22] Song J.L., Liu W.Y., Meng L., Liu X., Zhao Y. (2014). Development of large vertical high pressure gas quenching vacuum furnace. Met. Work..

[23] Wang Q., Jiang Z., Shen Z.Y., Lu J. (2012). Improvement on vacuum heat treatment process for die steel. Heat Treat..

[24] Yu L., Liu Q.S., Yang Y. (2012). A New Type of Fins Heat Exchanger Working for High-pressure Gas Quenching Furnace. Press. Vessel Technol..

[25] Shanghai Automobile Transmission Co., Ltd. (2012). Gas Carburizing and Quenching Process for Internal Gear Ring of Transmission: CN102808188B.

[26] Yu X.F., Wang S.Y., Wang Y.P., Yang S., Yang Y., Su Y., Feng X. (2022). Effect of Vacuum Graded Quenching on Microstructure and Mechanical Properties of 8Cr4Mo4V Steel. Chin. J. Mater. Res..

[27] Heuer V., Faron D.R., Bolton D., Lifshits M., Loeser K. (2013). Distortion Control of Transmission Components by Optimized High Pressure Gas Quenching. J. Mater. Eng. Perform..

[28] Pan Z. (2021). High pressure gas quenching equipment and technology. Heat Treat. Met..

[29] Yu T., Tan Z.L., Wang J., Zhang M. (2022). Realization of quenching & dynamic partitioning on large-size parts. Mater. Manuf. Processes.

[30] Zhang W.M. (2001). Effect of quenching method on quenching structure and tempering hardness of cold-working die steel SKD11. Heat Treat. Technol. Equip..

[31] Lin Z.B. (2015). Study on Vacuum Quenching Process of Cr12MoV Steel. Master’s Thesis.

[32] Yu H.Y., Zhao Z.W., Fan S.H. (1989). Microstructure and hardness of vacuum air quenched high speed steel. Shanghai Met..

[33] Wang Y.L., Wang J. (2009). Comparison of Microstructure and Properties of 1Cr17Ni2 Steel between Vacuum Gas Quenching and Conventional Oil Quenching. Hot Work. Technol..

[34] Guanghua Y., Xinmin H., Yanqing W., Xingguo Q., Ming Y., Zuoming C., Kang J. (2010). Effects of Heat Treatment On Mechanical Properties Of H13 Steel. Met. Sci. Heat Treat..

[35] Zhang J.G., Cong P.W. (2005). Vacuum Heat Treatment of Hot-Work Die Steel H13. Heat Treat. Met..

[36] Zhou A.Q. (2003). Effect of Isothermal Spheroidization on Microstructure and Properties of Steel H13. Heat Treat. Met..

[37] Ye J., Lu J.M. (2003). Materials for Large-sized Die-Casting Dies and its Vacuum Heat Treatment Technology. Heat Treat. Met..

[38] Syifa L., Ahmad F., Bambang S.I. (2019). The Effect of Vacuum Quenching on Corrosion and Hardness of the Surface of SKD61 Steel. IOP Conference Series: Materials Science and Engineering.

[39] Sola R., Giovanardi R., Veronesi P., Poli G. (2013). Effect of quenching method on the wear and corrosion resistance of stainless steel AISI 420 (TYPE 30Kh13). Met. Sci. Heat Treat..

[40] Macchion O., Zahrai S., Bouwman J.W. (2006). Heat transfer from typical loads within gas quenching furnace. J. Mater. Process. Technol..

[41] Thuvander A., Melander A., Lind M., Lior N., Bark F. Prediction of convective heat transfer coefficients and examination of their effects on distortion of tubes quenched by gas cooling. Proceedings of the 4th ASM Heat Treatment and Surface Engineering Conference in Europe.

[42] Igarashi T. (1981). Characteristics of the flow around two circular cylinders arranged in tandem: 1st report. Bull. JSME.

[43] Roshko A. (1955). On the wake and drag of bluff bodies. J. Aeronaut. Sci..

[44] Roshko A. (1993). Perspectives on bluff body aerodynamics. J. Wind. Eng. Ind. Aerodyn..

[45] Koenig K., Roshko A. (1985). An experimental study of geometrical effects on the drag and flow field of two bluff bodies separated by a gap. J. Fluid Mech..

[46] Bless F., Edenhofer B. (1997). Advances in increasing the quenching speed of single-chamber vacuum furnaces with high-pressure gas-quench systems. Heat Treat. Met..

[47] Pritchard J.E., Nurnberg G., Shoukri M. (1996). Computer modelling of pressure gas quenching in vacuum furnaces. Heat Treat. Met..

[48] Su X.W., Gu M. (2008). Research status and prospects of the numerical simulation of quenching process. Heat Treat. Met..

[49] Kula P., Korecki M., Pietrasik R., Wołowiec E., Dybowski K., Kołodziejczyk Ł., Atraszkiewicz R., Krasowski M. (2009). FineCarb—The flexible system for low-pressure carburizing. New options andperformance. Jpn. Soc. Heat Treat..

[50] Dowling W., Pattok T., Ferguson B.L. (1997). Development of a carburizing and quenching simulation tool: Program overview. HTM J. Heat Treat. Mater..

[51] Pan J., Li Y., Li L. (2002). The application of computer simulation in the heat-treatment process of a large-scale bearing roller. Mater. Process. Technol..

[52] Song R.G., Zhang Q.Z. (2001). Heat treatment optimization for 7175 aluminum alloy by genetic algorithm. Mater. Sci. Eng. C.

[53] Song R.G., Zhang Q.Z. (2001). Heat treatment technique optimization for 7175 aluminium alloy by an artificial network and a genetic algorithm. Mater. Process. Technol..

[54] Xu L., Wang Z.J. (2012). Applicability of the turbulence model in numerical simulation of vacuum gas quenching process. Vacuum.

[55] Jin L.Y., Wang C.J. (2012). Safety Design and Analysis of Vacuum Gas-quenching Furnace. Equip. Electron. Prod. Manuf..

[56] Wang Q., Lu J., Sun W.C., Shen Y.W., Wang W. (2014). Numerical Simulation and Verification of Quenching Processes in Two Types of High Pressure Gas Quenching Vacuum Furnace. Heat Treat..

[57] Kumhar K.M., Rahul B., Sathyaseelan S., Babu K. (2023). Estimation and analysis of surface heat flux during gas quenching through IHC method. Mater. Today Proc..

[58] Wang T., Cong P.W., Li Y., Du C.H. (2018). Application of response surface methodology in structural optimization of gas quenching. Heat Treat. Met..

[59] Xu Z.X., Su X.L., Xu Q.Y., Liu B.C. (2016). Numerical simulation on vacuum solution heat treatment and gas quenching process of a low rhenium-containing Ni-based single crystal turbine blade. China Foundry.

[60] Yuan K.Y., Zhou L.H., Li J.W., Min Y.M., Liu L. (2018). Numerical Simulation of Vacuum Gas Quenching Process of Large Aircraft Thin-wall Part. Shanghai Met..

[61] Sugianto A., Narazaki M., Kogawara M., Shirayori A., Kim S.Y., Kubota S. (2009). Numerical simulation and experimental verification of carburizing-quenching process of SCr420H steel helical gear. J. Mater. Process. Technol..

[62] Cheng H.M., Wang H.G., Xie J.B. (2006). Calculation of coupled problem between temperature and phase transformation during gas quenching in high pressure. Appl. Math. Mech. Engl. Ed..

[63] Cheng H., Huang X., Fan J., Wang H. (1999). The application of rational approximation in the calculation of a temperature field with a non-linear surface heat-transfer coefficient during quenching for 42CrMo steel cylinder. Met. Mater. Int..

[64] Hao X.W., Zhang W.M., Chen N.L., Zuo X.W. (2007). 3-D Numerical Simulation of Heat Transfer Process in Vacuum Heat Treatment Furnace. Heat Treat. Met..

[65] Schmidt F.F., Fritsching U. (2009). Homogenisation of heat treatment process in high pressure gas quenching. Int. Heat Treat. Surf. Eng..

[66] Lior N. (2004). The cooling process in gas quenching. J. Mater. Process. Technol..

[67] Luo Y., Kang J.W., Liu B.C., Rong Y.M. (2007). Numerical Simulation of Gas Quenching Process of Workpiece. Hot Work. Technol..

[68] Yazdi A.Z., Sajjadi S.A., Zebarjad S.M., Nezhad S.M. (2008). Prediction of hardness at different points of Jominy specimen using quench factor analysis method. J. Mater. Process. Technol..

[69] Flynn R.J., Robinson J.S. (2004). The application of advances in quench factor analysis property prediction to the heat treatment of 7010 aluminium alloy. J. Mater. Process. Technol..

[70] Kianezhad M., Sajjadi S.A. (2013). Improvement of Quench Factor Analysis in Phase and Hardness Prediction of a Quenched Steel. Metall. Mater. Trans..

[71] Kianezhad M., Sajjadi S.A., Vafaeenezhad H. (2015). A Numerical Approach to the Prediction of Hardness at Different Points of a Heat-Treated Steel. J. Mater. Eng. Perform..

[72] Zhang K.Y., Dong W.C., Lu S.P. (2022). Experimental and Numerical Investigation of Stress and Distortion in AF1410 Steel Under Varying Quenching Conditions. J. Mater. Eng. Perform..

[73] Zhang S.Q., Wang Q.S. (2005). Present situation and development trend of positive pressure gas quenching. Heat Treat. Technol. Equip..

